# Supplementary material for: Perceptual fusion of musical notes by native Amazonians suggests universal representations of musical intervals
Source: Nat Commun. 2020 Jun 3;11:2786. doi: 10.1038/s41467-020-16448-6 (PMC7270137; doi:10.1038/s41467-020-16448-6)
Supplement: Supplementary file 1 — Supplementary Information [file 41467_2020_16448_MOESM1_ESM.pdf]

Supplementary Information

Perceptual fusion of musical notes by native Amazonians suggests universal representations of musical intervals

McPherson et al.

Interval Stimuli

	Just Intonation		Equal Temperament	
Interval Name	Ratio	Interval in cents	Semitones	Interval in Cents
Unison	1:1	0	0	0
Major Second	9:8	203	2	200
Major Third	5:4	386	4	400
Perfect Fourth	4:3	498	5	500
Tritone (Aug. 4th/Dim 5th)	45:32	590	6	600
Perfect Fifth	3:2	702	7	700
Major Seventh	15:8	1088	11	1100
Octave	1:2	1200	12	1200
Minor Ninth	32:15	1312	13	1300

Supplementary Table 1. Interval and Chord Stimuli. Table of intervals used in fusion and preference experiments (as f0 ratios for Just Intonation, and in semitones for Equal Temperament, with sizes in cents in both cases to aid comparison).

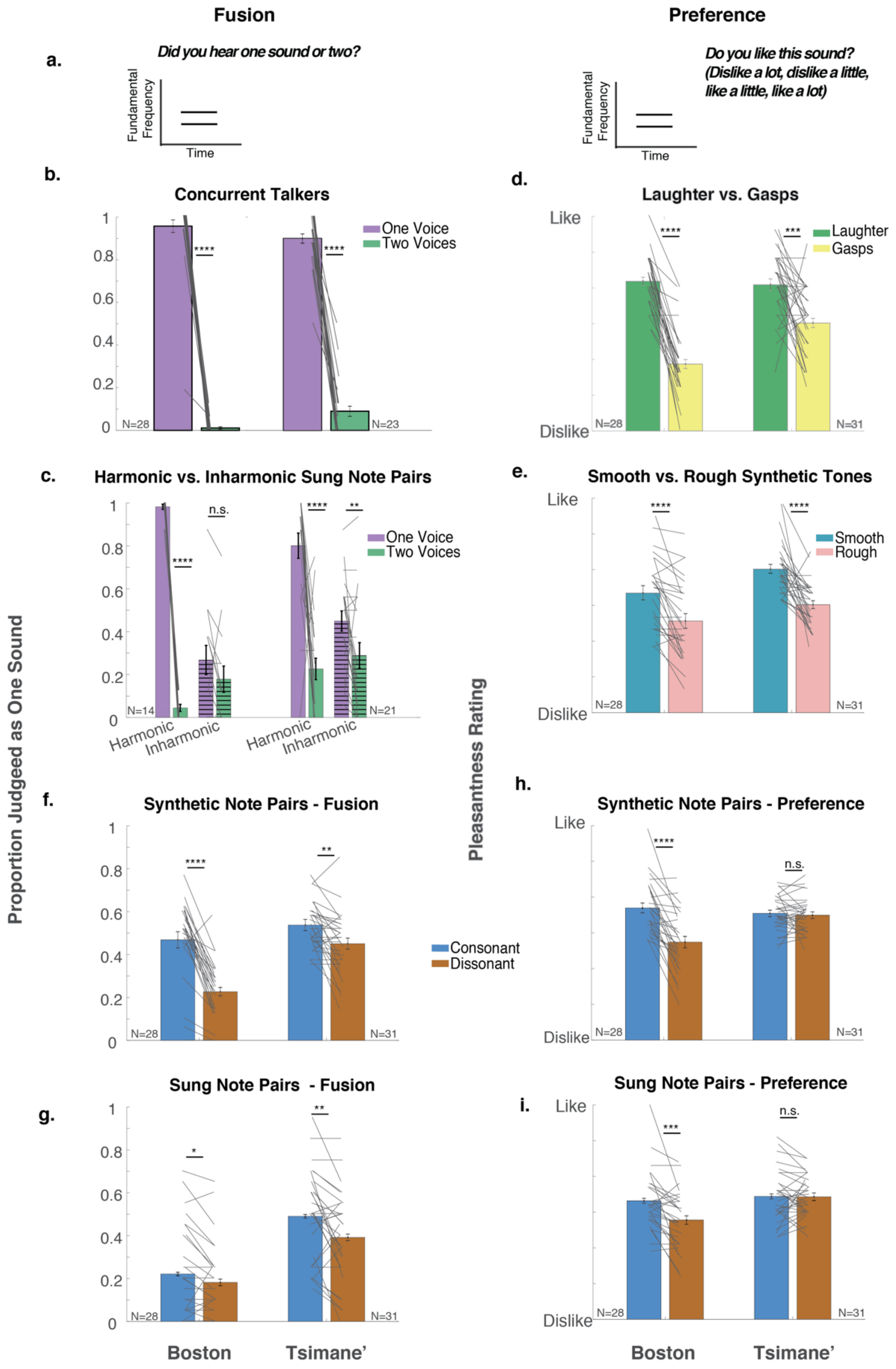

Supplementary Figure 1: Summary of results of in-person experiments. This figure is identical to the main Figure 2, but with the addition of data for individual participants, represented by lines. a) Schematic of trial structure for fusion (left) and preference (right) experiments. Participants heard a stimulus, and judged whether it contained one or two sounds, or rated its pleasantness. Line

segments denote individual notes, as were presented in the main experiments with musical intervals. b) Results of first fusion control experiment, in which participants heard one or two concurrent talkers. Not all Tsimane' participants completed the experiment (hence the smaller N compared to other panels). Here and in c, f and g, graph plots proportion of trials on which participants reported hearing one sound, plotted separately for Boston non-musicians and Tsimane'. Here and in c-h, plots show the mean +/- SEM. Results for individual participants are shown in Supplementary Figure 1. c) Results of second fusion control experiment, in which a separate set of participants heard one or two concurrent sung vowels, resynthesized to be either harmonic or inharmonic. F0 difference between vowels was chosen to avoid fusion in Western listeners when the notes were harmonic. Participants for this experiment were different from those for other experiments (hence different sample size). d-e) Results of preference control experiments, in which participants rated the pleasantness of recorded laughs and gasps, and of smooth and rough synthetic tones, respectively. In the latter case, tones consisted of pairs of frequencies presented either dichotically, to avoid beating, or diotically, to produce beating (roughness). f) Results of fusion experiment with musical intervals composed of synthetic notes. Fusion judgments were pooled across canonically consonant and dissonant musical intervals (and across tuning systems, which gave indistinguishable results). g) Results of fusion experiment with sung notes, pooled across consonant and dissonant intervals. Here and in (i), plots show the mean +/- within-participant SEM. h) Results of preference experiments with musical intervals composed of synthetic notes (averaged within consonant and dissonant subsets, and tuning systems). i) Results of preference experiments with musical intervals composed of sung notes (averaged within consonant and dissonant subsets). Across all results graphs, asterisks denote statistical significance of pairwise comparisons: * $p<0.05$, ** $p<0.01$, *** $p<0.001$, **** $p<0.0001$, and n.s. not significant. For subplots b, c, f and g, two-sided Wilcoxon signed-rank tests were used to test for differences between conditions. For subplots d, e, h, i, two-tailed paired t-tests were used to test for differences between conditions.

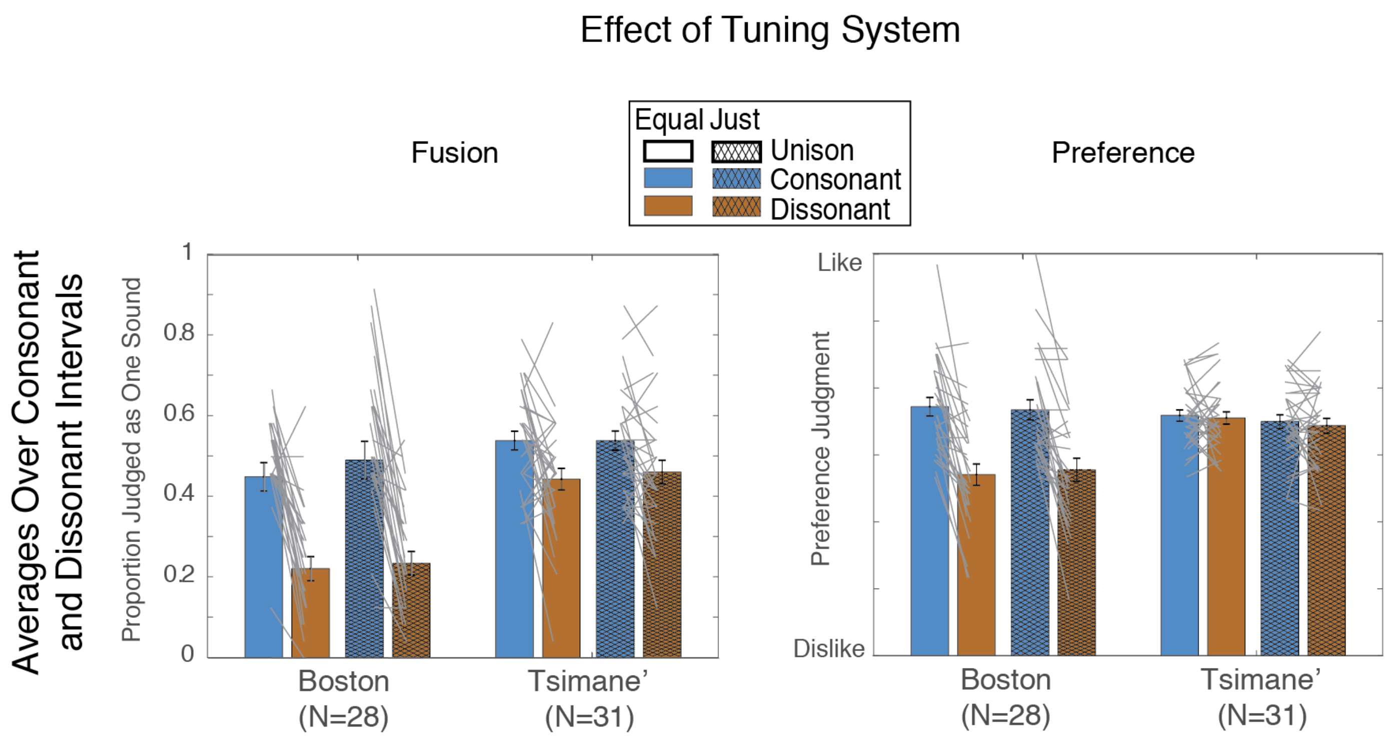

Supplementary Figure 2. Fusion and preference experiment results separated by tuning system. Results, averaged across consonant and dissonant intervals, but separated by tuning system. Bars plot mean values +/- SEM. Lines plot results for individual participants.

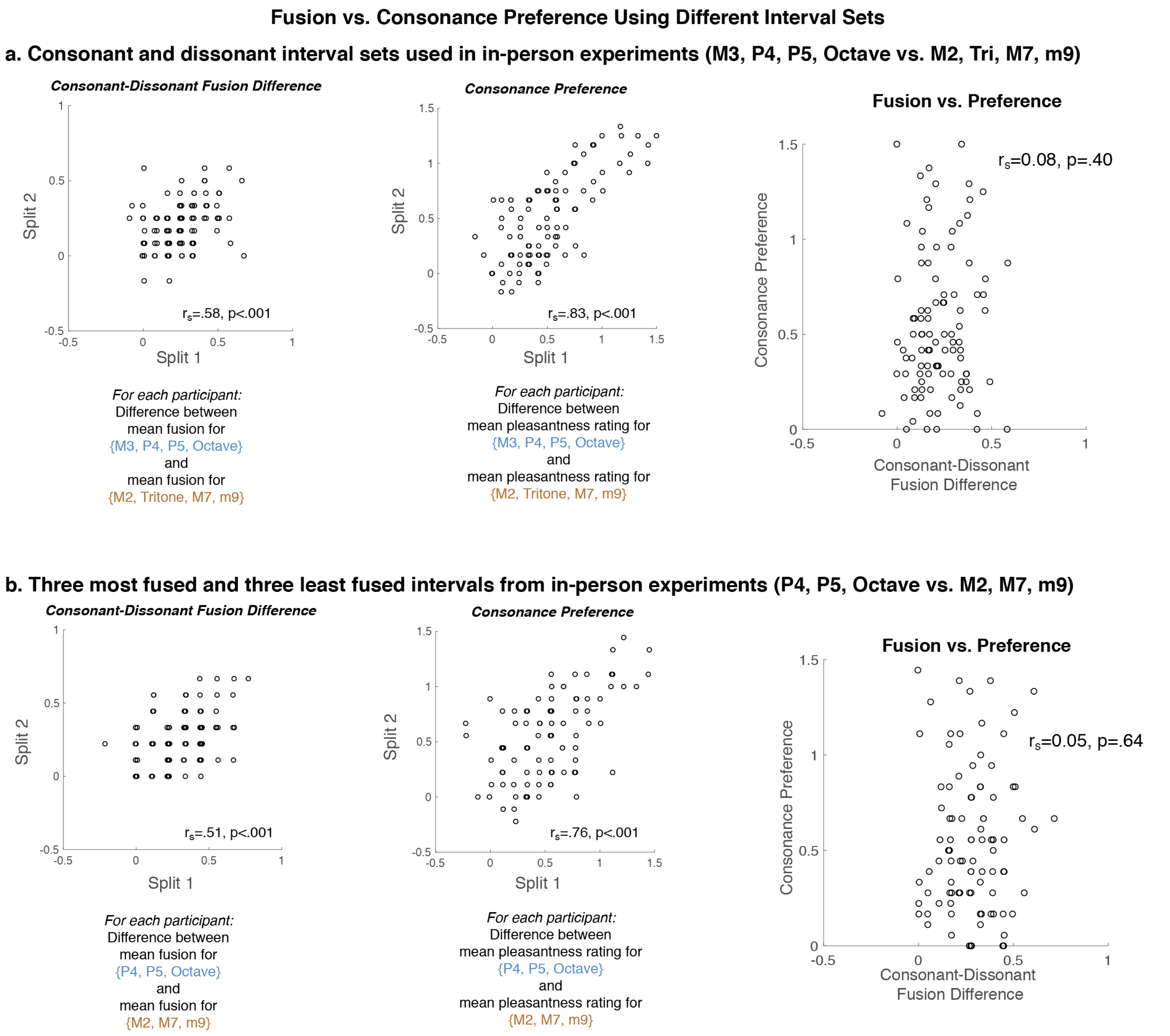

Supplementary Figure 3. Alternative analyses of individual differences in fusion and consonance preferences (using alternative consonant and dissonant interval sets). a. Analyses from Figure 4d-f repeated using the four consonant and four dissonant intervals from the main in-lab studies. b. Analyses from Figure 4d-f repeated using the three most- and least-fused intervals from main in-lab studies. Left two panels (a-b): test-retest reliability of the difference in fusion and pleasantness for consonant and dissonant intervals, computed from even and odd trials. Reliabilities were Spearman-Brown corrected to best estimate reliabilities of measures derived from full experiment. Right panel (a-b): consonance-dissonant pleasantness difference vs. consonant-dissonant fusion difference for individual participants. Dots represent individual participants. In all three panels, individual dots were jittered by a small amount to mitigate the visual effect of dot overlap.
