# Supplementary material for: Perceptual fusion of musical notes by native Amazonians suggests universal representations of musical intervals
Source: Nat Commun. 2020 Jun 3;11:2786. doi: 10.1038/s41467-020-16448-6 (PMC7270137; doi:10.1038/s41467-020-16448-6)
Supplement: Supplementary file 3 — Reporting Summary [file 41467_2020_16448_MOESM3_ESM.pdf]

nature research

Corresponding author(s): Malinda McPherson and Josh McDermott
Last updated by author(s): Apr 11, 2020

Reporting Summary

Nature Research wishes to improve the reproducibility of the work that we publish. This form provides structure for consistency and transparency in reporting. For further information on Nature Research policies, see Authors & Referees and the Editorial Policy Checklist.

Statistics

For all statistical analyses, confirm that the following items are present in the figure legend, table legend, main text, or Methods section.

n/a	Confirmed	
☐	☒	The exact sample size (*n*) for each experimental group/condition, given as a discrete number and unit of measurement
☐	☒	A statement on whether measurements were taken from distinct samples or whether the same sample was measured repeatedly
☐	☒	The statistical test(s) used AND whether they are one- or two-sided<br>*Only common tests should be described solely by name; describe more complex techniques in the Methods section.*
☐	☒	A description of all covariates tested
☐	☒	A description of any assumptions or corrections, such as tests of normality and adjustment for multiple comparisons
☐	☒	A full description of the statistical parameters including central tendency (e.g. means) or other basic estimates (e.g. regression coefficient) AND variation (e.g. standard deviation) or associated estimates of uncertainty (e.g. confidence intervals)
☐	☒	For null hypothesis testing, the test statistic (e.g. *F*, *t*, *r*) with confidence intervals, effect sizes, degrees of freedom and *P* value noted<br>*Give P values as exact values whenever suitable.*
☒	☐	For Bayesian analysis, information on the choice of priors and Markov chain Monte Carlo settings
☐	☒	For hierarchical and complex designs, identification of the appropriate level for tests and full reporting of outcomes
☐	☒	Estimates of effect sizes (e.g. Cohen's *d*, Pearson's *r*), indicating how they were calculated

Our web collection on statistics for biologists contains articles on many of the points above.

Software and code

Policy information about availability of computer code

Data collection	MATLAB (2018b) was used to play stimuli and record participant responses.
Data analysis	For data analysis we used standard MATLAB (2018b) tools (mean, standard deviation, t-tests, correlations, etc), as well as SPSS (Version 25).

For manuscripts utilizing custom algorithms or software that are central to the research but not yet described in published literature, software must be made available to editors/reviewers. We strongly encourage code deposition in a community repository (e.g. GitHub). See the Nature Research guidelines for submitting code & software for further information.

Data

Policy information about availability of data

All manuscripts must include a data availability statement. This statement should provide the following information, where applicable:
- Accession codes, unique identifiers, or web links for publicly available datasets
- A list of figures that have associated raw data
- A description of any restrictions on data availability

The authors declare that the data supporting the findings of this study are available within the supplementary information files. The RWC Instrument Database can be downloaded at https://staff.aist.go.jp/m.goto/RWC-MDB/. The recorded vocalization stimuli used in this study are available from the authors upon request.

Field-specific reporting

Please select the one below that is the best fit for your research. If you are not sure, read the appropriate sections before making your selection.

☐ Life sciences ☒ Behavioural & social sciences ☐ Ecological, evolutionary & environmental sciences

For a reference copy of the document with all sections, see nature.com/documents/nr-reporting-summary-flat.pdf

Behavioural & social sciences study design

All studies must disclose on these points even when the disclosure is negative.

Study description

This quantitative study examines psychophysical judgments and pleasantness ratings in response to a set of sounds. We tested three different participant groups (Boston non-musicians, indigenous Bolivians, and participants from Canada and the U.S.A. recruited online) to examine how responses would vary across different cultures and individuals.

Research sample

We used two sets of Boston participants and two sets of Tsimane' participants, and one set of online participants:
Set 1, Boston: 28 participants (14 female, mean age=33.7, years, S.D.= 8.9 years)
Set 2, Boston: 14 participants (8 female, mean age= 31.9, S.D.=8.8 years)
Set 1, Tsimane': 31 participants (14 female, mean age of 23.4 years, S.D.=5.7 years)
Set 2, Tsimane': 21 participants (5 female, mean age= 26.8 years, S.D.=8.9 years)

Set 1 completed all experiments except for the the fusion control experiment with harmonic and inharmonic concurrent voices (Figure 2c). Set 2 completed only this control experiment.

Online Experiment: 140 participants (43 female, mean age = 38.2 years, S.D.=10.3 years)

Sample sizes for Boston were chosen based on power analyses of pilot fusion judgment data with synthetic note intervals. We measured the split-half reliability of the pattern of fusion across all intervals, averaged across different numbers of participants. We chose a sample size expected to yield a split-half reliability exceeding r=.95, 90% of the time (18 participants).

Sample sizes for the Tsimane' were as large as possible given practical constraints and were larger than the Boston sample in each case.

Sample size for the online experiment was chosen so that we could detect a correlation of r=.3 between fusion and preference measures from the two experiments with a significance threshold of p<.05, 90% of the time. This yielded a target sample size of 92 participants. We ran ~50% more than this number in order to be able to exclude non-compliant participants.

Sampling strategy

We used convenience sampling. Bolivian participants were recruited by word of mouth (we often were able to recruit all eligible adults within individual Tsimane' villages). Boston participants were recruited using online recruiting platforms such as department-administered recruitment email lists and Craigslist. Online participants were recruited on Amazon's Mechanical Turk platform, using a geographic filter to restrict participation to individuals with IP addresses in the United States or Canada.

Data collection

Stimuli were played by MacBook Air laptop computers using over-ear closed headphones (Sennheiser HD 280Pro), at a presentation level of 70 dB SPL. The headphones used are designed to attenuate ambient noise (by up to 32 dB depending on the frequency) and are thus well suited for experiments in outdoor or public settings. Instructions and responses were in English in Boston, and in Tsimane' for all Tsimane' participants. For Tsimane' participants, translators (who spoke Tsimane' and Spanish) delivered the instructions and interpreted the participants' verbal responses. Experimenters were trained to recognize the Tsimane' words for the response options so they could evaluate the correctness of the translator's response. The experimenter entered the spoken response into the MATLAB interface. Experimenters were blind to the stimuli being presented, and to the correctness of participant's responses (for the fusion experiments), to avoid biasing responses and data entry.

Online participants were recruited through Amazon's Mechanical Turk platform, using a geographic filter to restrict participation to individuals logging on from the United States or Canada. Participants began by completing a demographic survey followed by a brief 'headphone check' experiment intended to help ensure that they were wearing headphones or earphones. Participants completed the study at their own computer and entered their own data.

Timing

Experiments were conducted in August 2019 (Tsimane'), September- November 2019 (USA), and November 2019 (Online). In person experiments were conducted in Tsimane' villages (for Tsimane' participants), and in public spaces on or near the MIT campus (for Boston participants).

Data exclusions

In-Person Experiments: One Tsimane' participant was removed from the analyses because they appeared to reverse the instructions on the first control experiment.

Online Experiment: We included for analysis all participants with 0 years of self-reported musical experience who performed at ceiling on the control experiment (which here was intended to identify non-compliant or inattentive online participants). 47 participants were excluded. The rationale for excluding participants based on musicianship and the control experiment was established a-priori: because the Tsimane' we tested have little musical training, we sought non-musicians to establish a matched group for comparison. We ran the control experiment with the aim of using it to filter participants.

Non-participation	10 Tsimane' participants began the experiments but did not complete them for various reasons (e.g., restless or sick children who needed attention, or noncompliance).
Randomization	All participants run in person completed the same set of studies, but the order of those studies were randomized within participant. Likewise, all participants run online completed the same set of studies, but the order of those studies were randomized within participant.

Reporting for specific materials, systems and methods

We require information from authors about some types of materials, experimental systems and methods used in many studies. Here, indicate whether each material, system or method listed is relevant to your study. If you are not sure if a list item applies to your research, read the appropriate section before selecting a response.

Materials & experimental systems

n/a	Involved in the study
☒	☐ Antibodies
☒	☐ Eukaryotic cell lines
☒	☐ Palaeontology
☒	☐ Animals and other organisms
☐	☒ Human research participants
☒	☐ Clinical data

Methods

n/a	Involved in the study
☒	☐ ChIP-seq
☒	☐ Flow cytometry
☒	☐ MRI-based neuroimaging

Human research participants

Policy information about studies involving human research participants

Population characteristics	See above.
Recruitment	Bolivian participants were recruited by word of mouth, and Boston participants were recruited using online recruiting platforms such as department-administered subject recruitment email lists and Craigslist, in order to get representative samples within each group. Online participants were recruited on Amazon's Mechanical Turk platform, using a geographic filter to restrict participation to individuals with IP addresses in the United States or Canada. During recruitment, participants were only told that the study would involve listening to sounds over headphones and making basic judgments about them. The description of the experiment was intentionally vague to reduce self-selection bias during recruitment. We often were able to recruit all eligible adults within individual Tsimane' villages, such that the sample is likely to be representative of the general Tsimane' population.
Ethics oversight	The study was approved by the Tsimane' Council (the governing body of the Tsimane' in the Maniqui basin, where the experiments took place), and the Committee on the Use of Humans as Experimental Subjects at MIT.

Note that full information on the approval of the study protocol must also be provided in the manuscript.
