# Supplementary material for: Perceptual fusion of musical notes by native Amazonians suggests universal representations of musical intervals
Source: Nat Commun. 2020 Jun 3;11:2786. doi: 10.1038/s41467-020-16448-6 (PMC7270137; doi:10.1038/s41467-020-16448-6)
Supplement: Supplementary file 4 — Source Data [file 41467_2020_16448_MOESM4_ESM.zip › Data_McPherson_etal_PerceptualFusion/README.rtf]

Data for “Perceptual fusion of musical notes by native Amazonians suggests universal representations of musical intervals”Authors: Malinda J. McPherson, Sophia E. Dolan, Alex Durango, Tomas Ossandon, Joaquin Valdes, Eduardo A. Undurraga, Nori Jacoby, Ricardo A. Godoy, Josh H. McDermottCorresponding Author: Malinda J. McPherson - mjmcp@mit.eduData organized by figure panel; columns of data correspond to bars on bar plots or x/y coordinates for scatterplots. For example, Fig2bBoston.csv contains the data from Boston participants, first column is ‘One Voice’ condition, second column is ‘Two Voices’ condition. Fig2cBolivia.csv contains data from Tsimane’ participants, first column is Harmonic (One Voice), second column is Harmonic (Two Voices), third column is Inharmonic (One Voice), fourth column is Inharmonic (Two Voices). 
